# Market capitalization shock effects on open innovation models in e-commerce: golden cut q-rung orthopair fuzzy multicriteria decision-making analysis

**DOI:** 10.1186/s40854-023-00461-x

**Published:** 2023-02-07

**Authors:** Nikita Moiseev, Alexey Mikhaylov, Hasan Dinçer, Serhat Yüksel

**Affiliations:** 1 Department of Mathematical Methods in Economics, Plekhanov Russian University of Economics, Moscow, Russia; 2 Financial University Under the Government of the Russian Federation, Moscow, Russia; 3 The School of Business, İstanbul Medipol University, Istanbul, Turkey

**Keywords:** E-commerce, Golden cut, Open innovation, Sustainable trade

## Abstract

This research paper analyzes revenue trends in e-commerce, a sector with an annual sales volume of more than 340 billion dollars. The article evaluates, despite a scarcity of data, the effects on e-commerce development of the ubiquitous lockdowns and restriction measures introduced by most countries during the pandemic period. The analysis covers monthly data from January 1996 to February 2021. The research paper analyzes relative changes in the original time series through the autocorrelation function. The objects of this analysis are Amazon and Alibaba, as they are benchmarks in the e-commerce industry. This paper tests the shock effect on the e-commerce companies Alibaba in China and Amazon in the USA, concluding that it is weaker for companies with small market capitalizations. As a result, the effect on estimated e-trade volume in the USA was approximately 35% in 2020. Another evaluation considers fuzzy decision-making methodology. For this purpose, balanced scorecard-based open financial innovation models for the e-commerce industry are weighted with multistepwise weight assessment ratio analysis based on q-rung orthopair fuzzy sets and the golden cut. Within this framework, a detailed analysis of competitors should be made. The paper proves that this situation positively affects the development of successful financial innovation models for the e-commerce industry. Therefore, it may be possible to attract greater attention from e-commerce companies for these financial innovation products.

## Introduction

Open innovation is innovative modeling that assumes firms can use external and internal resources for advancing product collaboration to achieve their aims. A key benefit of “open innovation” is that it reduces production expenses (Povolna 2019; Poon et al. 2020). The current research paper analyzes the financial parameters of the US e-commerce sector from 1996 to 2021. The relationship between revenue forecasts for companies based on prepandemic and postpandemic values illustrates the effects on the industry (MacKinlay 1997; Lukin 2019). This idea ties in with the paper’s goal—to evaluate the impact of restrictive measures introduced during the pandemic on the role of market capitalization in e-commerce industry development.

The sample data separately emphasize two individual firms in the analysis: Amazon and Alibaba. This approach is justified by the fact that these two companies are benchmarks and leaders of the global e-commerce sector in the strongest technological economies: the USA and China. At the end of 2020, Amazon accounted for 39% of all US e-commerce sales, while Alibaba had a 58.2% share of all retail e-commerce sales in mainland China (Nakhate and Jain 2020). The research methods presented in the analysis of Amazon and Alibaba are necessary to better understand the situation in the e-commerce industry.

The objective of this paper is to analyze the effects of market capitalization shocks on open innovation models in the e-commerce industry. The novelty is based on new data for making forecasts about e-commerce companies in the USA. So, the findings fill the gap in the structure of the e-commerce sector in China and the USA, which is much more complex, with a weaker shock effect for small-market-capitalization companies (market capitalizations from $300 million to $2 billion). The finding adds to the growing literature on the effects of market capitalization on open innovation models in the e-commerce sector. The results show the positive impact of expanding production networks in e-commerce networks and further develop the ideas of Acemoglu et al. (2016) and Carvalho and Gabaix (2013). Moreover, the effect of market capitalization on open innovation models in US e-commerce companies in recent years is also determined.

When analyzing the effects of market capitalization shocks on the e-commerce industry, companies should prioritize learning new financial ideas with competition and benchmarking. Within this framework, a detailed competitor analysis should be performed. With the help of this comprehensive analysis, creative ideas can be obtained. This situation has a positive impact on the development of successful financial innovation models for the e-commerce industry. Therefore, it may be possible to gain greater attention from e-commerce companies for these financial innovation products. Turan (2015), Akdere and Benli (2018), and Egorov and Pomazkin (2021) have highlighted the significance of creative ideas regarding the effectiveness of financial innovation models for the e-commerce industry.

At the same time, the study shows that the state should seek more control in the production chain to strengthen the supervision of large-cap e-commerce companies, as existing studies have proven for the US and South African companies (Adam 2020). New data allow a detailed analysis and forecast of e-commerce companies in the USA and the underlying macroeconomic effects (Atalay et al. 2011; Tintelnot et al. 2017; Oberfield 2018; Khan et al. 2021).

The novelty of the current study is the analysis of deglobalization effects amid the rapid development of e-commerce open innovation in the USA and China. In addition to this issue, another novelty of this study is developing evaluations by considering econometric models and a fuzzy decision-making methodology. This situation provides an opportunity to reach more precise results. Furthermore, appropriate strategies can be created to improve open financial innovation models for the e-commerce industry. The main finding is that appropriate strategies can be created to achieve these improvements. These findings add to the growing literature on the role of market capitalization for open innovation models in the e-commerce sector. Future research can explain how production networks spread impacts e-commerce networks exactly. This paper contributes to the literature on the effects of market capitalization for open innovation models on the e-commerce sector in the USA. This paper can prove the implications of the researchers make about market capitalization for open innovation models and deglobalization (Shea and Poast 2018; McCarthy 2019; Garcia-Sanchez and Garcia-Sanchez 2020).

The structure of the paper is as follows. The introduction includes a description of the research problem; the second section of the study includes a literature evaluation of e-commerce, open innovation, market capitalization shocks, and methods. The third section discusses the methodology. The fourth section explains the analysis results. The fifth and seventh sections focus on the discussions and conclusions of the study. The final two sections focus on the strengths and limitations of the study and its practical and theoretical implications.

## Literature review

### e-commerce

This subchapter is about e-commerce. It is a sphere of the digital economy, which includes all financial and trade transactions carried out using the computer networks and business processes associated with such transactions: electronic information exchange (electronic data interchange, or EDI), electronic capital movement (electronic funds transfer, or EFT), electronic commerce (English e-trade), electronic money (e-cash), electronic marketing (e-marketing), electronic banking (e-banking), and electronic insurance services (e-insurance).

Deglobalization influences the strong development of e-commerce open innovation in the USA: (1) The market capitalization for open innovation models on small-cap companies (market capitalization from $300 million to $2 billion) was weaker than on the large-cap companies (market capitalization value of more than $10 billion) because of creative strategies in social media marketing (Ashley and Tuten 2015) and field experimentation in marketing research. (2) The effects were weaker on small-cap companies than large companies because of theoretical foundations and required capabilities, as well as dynamic marketing capabilities (Barrales-Molina 2014; Samaha et al. 2014; Key and Czaplewski 2017).

Many articles have explained it via marketing theory (Carins and Rundle-Thiele 2014) and marketing automation. Expanding upon the idea of marketing and e-commerce, many articles have studied the factors influencing consumer behavior when shopping online. Several factors influence purchasing behavior, such as demographic factors, social factors, Internet and computer skills, website design, social networks, situational factors, product characteristics, promotions, payment options, and delivery of goods in online stores (West and Wood 2013; Park and Choi 2019; Dvoulety 2019; Povolna 2019; Alber 2020; Ayittey et al. 2020). Since the prevalence of coronavirus was measured by cumulative cases and cumulative deaths, researchers relied on data from the most affected countries. However, these effects have not been confirmed in Italy and the USA (Pandey and Parmar 2019; Nakhate and Jain 2020).

### Open innovation

This subchapter is about open innovation. Open innovation is a term for a business paradigm that provides, in contrast to the previously prevailing approaches, a more flexible policy regarding R&D and intellectual property. It is very popular in newer fields—cryptocurrencies, cashless payments, venture investments, roboadvizing, cloud investment computing, virtual reality, smart payments, and blockchain—and described in new research and review papers (Xiao and Ke 2021). Technological open innovation in the USA empowers value cocreation and can be divided into several types (Gawer 2014; Chang et al. 2015; Buhalis and Foerste 2015). Many US companies began to implement green open innovation in 2021 (Iansiti and Levien 2004).

Moreover, many research papers have studied the impact of innovation practices on sustainable small and medium enterprise performance (Asad et al. 2018). Researchers have found many success factors for small and medium enterprises with regard to market capitalization in open innovation models (Asad and Kashif 2021; Asif et al. 2021). Furthermore, the application of multicriteria methods in the study of capital markets is described in many papers. Balanced scorecard-based open financial innovation models of Brazilian and Indian stock markets have been studied in many papers. Ranking-based multicriteria decision-making (MCDM) models in financial management applications are suitable for such research.

During the market capitalization for open innovation models, for example, the construction methods of investment portfolios changed in various ways. Open innovation has also been expanding in the e-commerce industry. Many researchers studied the arrangements of open innovation in US e-commerce for marketing excellence (Moorman and Day, 2016). The papers found that US e-commerce open innovation is implemented via marketing (Kannan 2017). Several papers made marketing analytics for data-rich environments (Germann et al. 2015; Wedel and Kannan 2016). Regarding some noteworthy types of open innovation beneficial to e-commerce, the theory of ecosystems has been utilized in many research papers (Jacobides et al. 2018) by broadening the locus of value creation (Kapoor 2018). The sustained, superior performance of the business ecosystems (Kapoor and Agarwal 2017) of US companies was a proven result. The main questions are related to the organizational forms that shape new technology investments for coordinating and competing in ecosystems (Kapoor and Lee 2013) and the mechanism, with which platform participant strategies adapt for collective governance (O’Mahony and Karp 2017).

### Market capitalization shocks

This subchapter is about market capitalization shocks for open innovation. Under modern conditions, public industrial corporations have certain theoretical and applied tools that allow them to find free capital and increase profits thanks to the financial market. At the same time, public industrial corporations are subject to the negative impact of the rapid development of the financial market, expressed in periodic market shocks or so-called crisis phenomena. Several researchers determined the model’s prevalence on the industry indices of the Egyptian Stock Exchange from March to May 2020. They confirmed that the coefficient of determination between independent demand variables and a variable related to the e-commerce sector is 0.393. These findings are in good agreement with the International Monetary Fund’s assessment that even a milder flu pandemic shock could wipe out 0.5% of global gross domestic product, or approximately $300 billion (Elsayed and Elrhim 2020; Fernandes 2020).

Over the last 5 years, many researchers have found that the structure of the e-commerce sector is much more complex, and shock effects are weaker for companies with small market capitalizations (market capitalization from $300 million to $2 billion). Researchers have shown the positive impact of expanding production networks on e-commerce networks, developing the ideas of Acemoglu et al. (2016) and Carvalho and Gabaix (2013). Moreover, the effect of market capitalization on open innovation models generally in US e-commerce companies in recent years has also been determined.

Studies have also shown that since most products came from China and many industrial facilities were closed, no imports and exports of products occurred during the lockdown period. Some articles have aimed to identify the impact of shocks on e-commerce. In 2020, all delivery processes were hampered, which reduced the growth of e-commerce in some countries (Hasanat et al. 2020). Considering the research paper’s goal, the main hypothesis of the study is that the shock effect of market capitalization on open innovation models in the USA is stronger for large-cap companies (those with market capitalizations of over $10 billion) than small-cap companies (those with market capitalizations from $300 million to $2 billion). Much research has already tested this hypothesis using many types of data; this paper, meanwhile, uses updated data. The varied effects just mentioned could potentially be explained by spread production networks, which have a positive impact.

To test the presented hypothesis, the paper uses state space methods that have been used to study many data series (Rajan and Mathew 2012, Taveeapiradeecharoen et al. 2018). Another evaluation is performed using a fuzzy decision-making methodology. For this purpose, balanced scorecard-based open financial innovation models for the e-commerce industry are weighted with the multistepwise weight assessment ratio analysis (M-SWARA) method based on q-rung orthopair fuzzy sets (q-ROFSs) and the golden cut. With the help of this situation, the reliability of the findings can be checked.

### Golden cut q-rung orthopair fuzzy multicriteria decision-making analysis

This subchapter is about multicriteria methods, and a combination of q-ROFSs and M-SWARA are justified. MCDM is a subsector of operations research that explicitly evaluates multiple conflicting criteria in decision-making (both in everyday life and settings such as business, government, and medicine). The study uses and combines them as described in many papers (Mikhaylov et al. 2022; Shaikh et al. 2022). As for methodology literature, the major areas for time-varying parameter (TVP) regression with the Kalman filter are the dynamics of the volatility skew; impacts of factor price changes and technological progress on energy intensity; the time-varying parameter vector autoregressive model for economy and monetary policy; financial time series analysis in eco-friendly computing and communication systems; daily currency forecasting; and the price elasticity of electricity (Primiceri 2005; Marcellino et al. 2006; Koop et al. 2009; Bedendo and Hodges 2009; Nakajima et al. 2011, Rajan et al. 2012, Taveeapiradeecharoen et al. 2018; Tiwari and Menegaki 2019; Taveeapiradeecharoen and Aunsri 2020; Cooke 2020).

Many researchers have found that the golden cut q-rung orthopair fuzzy (q-ROF) MCDM analysis assumes that each share has a certain sensitivity to movements in the role of market capitalization for open innovation models on company revenue. They have found that shock effects drive company revenue in the e-commerce sectors of developed and emerging economies. This paper also develops ideas about the sustainability of shock effects (McDonagh and Prothero 2014) and the technological open innovation process in the USA based on value cocreation, which can be divided into several types (Iansiti and Levien 2004; Gawer 2014; Chang et al. 2015; Buhalis and Foerste, 2015).

## Methods

### Method of analysis and multicriteria decision-making analysis definitions

The methodology includes the major areas for TVP regression with the Kalman filter are dynamics of the volatility skew; the impact of factor price changes and technological progress on energy intensity; the time-varying parameter vector autoregressive model for economy and monetary policy; financial time series analysis in eco-friendly computing and communication systems; daily currency forecasting; price elasticity of electricity. The methodology is reproducible for new data sets. Operationalization of the variables is included. Structural breaks during the time series analysis are considered. To reduce the effect of structural changes, we use the Sup-LM test suggested using a bootstrap algorithm with fixed regressors (fixed regressor bootstrap) or a bootstrap based on residuals (residual bootstrap). However, they do not prove the asymptotic validity of bootstrap tests, limiting themselves to the derivation of the asymptotic distribution of test statistics and demonstration of bootstrap operation in simulations.

This method includes the following models: financial institution-oriented financial facilities (Alam et al. 2019; Dabrowski and Lottermoser 2019), customer interaction in financial issues (Poon et al. 2020; Al-Dmour et al. 2020), learning new financial ideas with competition and benchmarking (Chen 2018; Xiao and Ke 2021), organizational excellence in collaborative financial ideas (Liu et al. 2021; Meng et al. 2021). Financial innovation models can be based on financial institution-oriented financial facilities. Additionally, customer interaction in financial issues can be considered. Moreover, the financial innovation model includes linguistic scale criteria: no influence (n), somewhat influence (s), medium influence (m), high influence (h), and very high influence (vh). The methods of this paper are based on previous research using combinations of the golden cut q-ROF MCDM analysis (Yager 2016; Yang and Pang 2020). The main idea of q-ROF MCDM analysis is selection the best criterion from many variants.

### Data

The data include total e-trade volume in the USA from 1996 to 2021, uploaded from Federal Reserve Economic Data API (FRED) (Mikhaylov 2021). The paper uses a time series from 2004 to 2021 for Amazon because Amazon went public later. Back then, even the boldest optimists could not foresee that Amazon would eventually turn into one of the largest companies in the world. The Alibaba data set is chosen for the 2016–2021 period because Alibaba is a younger company. The large differences between the two data sets do not affect the results as in previous studies (Yager 2016; Yang and Pang 2020). The methods are consistent with the objectives in previous studies (Primiceri 2005; Bedendo and Hodges 2009; Nakajima et al. 2011). The methods highlight how objectives have been measured in many studies (Bedendo and Hodges, 2009; Nakajima et al. 2011).

As there is a difference in the years covered by the data taken for the two companies, we next provide some explanatory comments. When analyzing time series of different longitudes, it is necessary to solve various tasks related to studying the behavior of the observed parameter (for cyclicity, volatility, trends) and forecasting future values. The method of moving averages is used mainly for smoothing time series, which makes it possible to eliminate random fluctuations and reproduce values corresponding to the influence of the main factors. Rolling estimates are obtained by replacing a set of consecutive initial values of a time series within a selected time interval with arithmetic mean.

### Golden cut q-rung orthopair fuzzy multicriteria decision-making analysis

Yager (2016) created generalized orthopair fuzzy sets to make more efficient evaluations in MCDM analysis. This paper develops multicriteria methods and uses and combines q-ROFSs and M-SWARA.

The paper uses a methodology based on the most common TVP regression form:

1 $${y}_{t}={\beta }_{0,t}+{\beta }_{1,t}{x}_{1,t}+\dots +{\beta }_{k,t}{x}_{k,t}+{\nu }_{t}, {\nu }_{t}\sim N\left(0,{\sigma }_{\nu }^{2}\right)$$

2 $$\beta_{i,t + 1} = \beta_{i,t} + \xi_{it} ,{ }\xi_{it} \sim N\left( {0,\sigma_{i}^{2} } \right),{ }i = 0, \ldots ,k$$

where ${{\varvec{\beta}}}_{1}\sim N({\varvec{b}},{\varvec{P}})$, ${{\varvec{\eta}}}_{t}\sim iid N(0,{{\varvec{I}}}_{r})$, ${{\varvec{\varepsilon}}}_{t}\sim iid N(0,{{\varvec{I}}}_{N})$, $N$ is the number of modeled observations, ${{\varvec{T}}}_{t}$ is a parameters matrix for state transition, ${{\varvec{X}}}_{t}$ is the design matrix for the observation model, ${{\varvec{H}}}_{t}$ accounts for the state model’s error covariances, and ${{\varvec{G}}}_{t}$ accounts for the observation model’s error covariances. State space models are usually estimated using the Kalman filter (Kalman 1960); representing a recursive procedure of updating state estimates. Hence, this study gives a brief description of the Kalman filter algorithm. The predicted (a priori) state estimate and its covariance are computed below.

Such models are most conveniently estimated by state space methods. The general form of a state space model is as follows:

3 $$\begin{array}{*{20}c} {{\varvec{\beta}}_{t + 1} } \\ {m \times 1} \\ \end{array} = \begin{array}{*{20}c} {{\varvec{d}}_{t} } \\ {m \times 1} \\ \end{array} + \begin{array}{*{20}c} {{\varvec{T}}_{t} } \\ {m \times m} \\ \end{array} \times \begin{array}{*{20}c} {{\varvec{\beta}}_{t} } \\ {m \times 1} \\ \end{array} + \begin{array}{*{20}c} {{\varvec{H}}_{t} } \\ {m \times r} \\ \end{array} \times \begin{array}{*{20}c} {{\varvec{\eta}}_{t} } \\ {r \times 1} \\ \end{array}$$

4 $$\begin{array}{*{20}c} {{\varvec{y}}_{t} } \\ {N \times 1} \\ \end{array} = \begin{array}{*{20}c} {{\varvec{c}}_{t} } \\ {N \times 1} \\ \end{array} + \begin{array}{*{20}c} {{\varvec{X}}_{t} } \\ {N \times m} \\ \end{array} \times \begin{array}{*{20}c} {{\varvec{\beta}}_{t} } \\ {m \times 1} \\ \end{array} + \begin{array}{*{20}c} {{\varvec{G}}_{t} } \\ {N \times N} \\ \end{array} \times \begin{array}{*{20}c} {{\varvec{\varepsilon}}_{t} } \\ {N \times 1} \\ \end{array}$$

5 $$\hat{\user2{\beta }}_{t|t - 1} = {\varvec{d}}_{t} + {\varvec{T}}_{t} \hat{\user2{\beta }}_{t - 1|t - 1}$$

6 $${\varvec{P}}_{t|t - 1} = {\varvec{T}}_{t} {\varvec{P}}_{t - 1|t - 1} {\varvec{T}}_{t}^{T} + {\varvec{H}}_{t} {\varvec{H}}_{t}^{T}$$

Afterwards, a prefit residual and its covariance are computed:

7 $$\tilde{\user2{e}}_{t} = {\varvec{y}}_{t} - {\varvec{X}}_{t} \hat{\user2{\beta }}_{t|t - 1}$$

8 $${\varvec{S}}_{t} = {\varvec{X}}_{t} {\varvec{P}}_{t|t - 1} {\varvec{X}}_{t}^{T} + {\varvec{G}}_{t} {\varvec{G}}_{t}^{T}$$

Then the Kalman gain will be

9 $${\varvec{K}}_{t} = {\varvec{P}}_{t|t - 1} {\varvec{X}}_{t}^{T} {\varvec{S}}_{t}^{ - 1}$$

which is used to update the state estimate and its covariance matrix:

10 $$\hat{\user2{\beta }}_{t|t} = \hat{\user2{\beta }}_{t|t - 1} + {\varvec{K}}_{t} \tilde{\user2{e}}_{t}$$

11 $${\varvec{P}}_{t|t} = \left( {{\varvec{I}}_{t} - {\varvec{K}}_{t} {\varvec{X}}_{t} } \right){\varvec{P}}_{t|t - 1}$$

To perform the Kalman filtering procedure, one needs to define the set of initial parameters, which are: ${{\varvec{H}}}_{t}$, ${{\varvec{G}}}_{t}$, ${{\varvec{d}}}_{t}$, ${{\varvec{T}}}_{t}$, ${\widehat{{\varvec{\beta}}}}_{0|0,}$ and ${{\varvec{P}}}_{0|0}$. In this case, the following can be assumed: ${{\varvec{T}}}_{t}={{\varvec{I}}}_{m}$, which means that a priori, the consecutive change in each parameter depends solely on its previous value. The study also assumes that ${{\varvec{G}}}_{t}={\sigma }_{\nu }^{2}$ since the model has just one time series at a time. Unknown initial parameters are fitted by the Monte Carlo Markov chain method.

### Q-rung orthopair fuzzy set and multistepwise weight assessment ratio analysis approaches

As a result of this algorithm, the dynamics of each model parameter and its variance were obtained. This allows confidence intervals to be built and conclusions to be drawn about the evolution of the analyzed time series.

Atanassov (1983) generated intuitionistic fuzzy sets (IFSs) by considering membership and nonmembership degrees (${\mu }_{I}$, ${n}_{I}$), as in Eq. (12). The condition is defined as $0\le {\mu }_{I}\left(\vartheta \right)+{n}_{I}\left(\vartheta \right)\le 1$:

13 $$I=\left\{\langle {\vartheta ,\mu }_{I}(\vartheta ),{n}_{I}(\vartheta )\rangle /\vartheta \epsilon U\right\}$$

Yager (2016) developed Pythagorean fuzzy sets (PFSs) by new degrees (${\mu }_{P}$, ${n}_{P}$) with Eq. (13):

13 $$P=\left\{\langle {\vartheta ,\mu }_{P}(\vartheta ),{n}_{P}(\vartheta )\rangle /\vartheta \epsilon U\right\}$$

The condition is demonstrated in Eq. (14):

14 $$0\le {\left({\mu }_{P}(\vartheta )\right)}^{2}+{\left({n}_{P}(\vartheta )\right)}^{2}\le 1$$

Yager (2016) introduced q-ROFSs with new grades (${\mu }_{Q}$, ${n}_{Q}$) as in Eq. (15). In this process, the extension of IFSs and PFSs is considered:

15 $$Q=\left\{\langle {\vartheta ,\mu }_{Q}(\vartheta ),{n}_{Q}(\vartheta )\rangle /\vartheta \epsilon U\right\}$$

Equation (16) indicates the condition

16 $$0\le {\left({\mu }_{Q}(\vartheta )\right)}^{q}+{\left({n}_{Q}(\vartheta )\right)}^{q}\le 1, q\ge 1$$

The indeterminacy degree is shown in Eq. (17):

17 $${\pi }_{Q}\left(\vartheta \right)={\left({\left({\mu }_{Q}\left(\vartheta \right)\right)}^{q}+{\left({n}_{Q}\left(\vartheta \right)\right)}^{q}-{{\left({\mu }_{Q}\left(\vartheta \right)\right)}^{q}\left({n}_{Q}\left(\vartheta \right)\right)}^{q}\right)}^{1/q}$$

Calculations are made using Eqs. (18)–(21):

$${Q}_{1}=\left\{\langle {\vartheta ,{Q}_{1}(\mu }_{{Q}_{1}}(\vartheta ),{n}_{{Q}_{1}}(\vartheta ))\rangle /\vartheta \epsilon U\right\}$$ and $${Q}_{2}=\left\{\langle {\vartheta ,{Q}_{2}(\mu }_{{Q}_{2}}(\vartheta ),{n}_{{Q}_{2}}(\vartheta ))\rangle /\vartheta \epsilon U\right\}$$

18 $${Q}_{1}{\oplus Q}_{2}={\left({\left({\mu }_{{Q}_{1}}^{q}+{\mu }_{{Q}_{2}}^{q}-{\mu }_{{Q}_{1}}^{q}{\mu }_{{Q}_{2}}^{q}\right)}^{1/q}, {n}_{{Q}_{1}}{n}_{{Q}_{2}}\right)}$$

19 $${Q}_{1}{\otimes Q}_{2}={\left({\mu }_{{Q}_{1}}{\mu }_{{Q}_{2}}, {\left({n}_{{Q}_{1}}^{q}+{n}_{{Q}_{2}}^{q}-{n}_{{Q}_{1}}^{q}{n}_{{Q}_{2}}^{q}\right)}^{1/q}\right)}$$

20 $$\lambda Q={\left({\left(1-{\left(1-{\mu }_{Q}^{q} \right)}^{\lambda }\right)}^{1/q}, {\left({n}_{Q}\right)}^{\lambda }\right),\lambda >0}$$

21 $${Q}^{\lambda }={\left( {\left({\mu }_{Q}\right)}^{\lambda }, {\left(1-{\left(1-{n}_{Q}^{q} \right)}^{\lambda } \right)}^{1/q}\right),\lambda >0}$$

Equation (22) is used for defuzzification:

22 $$S\left(\vartheta \right)={\left({\mu }_{Q}(\vartheta )\right)}^{q}-{\left({n}_{Q}(\vartheta )\right)}^{q}$$

Golden cut ($\varphi$) is used in this study to define degrees (${\mu }_{G}$, ${n}_{G}$). Equations (23)–(25) are considered in this process. In these equations, *a* and *b* identify large and small quantities:

23 $$\varphi =\frac{a}{b}$$

24 $$\varphi =\frac{1+\sqrt{5}}{2}=1.618\dots$$

25 $$\varphi =\frac{{\mu }_{G}}{{n}_{G}}$$

Equations (26) and (27) adapt the golden cut to q-ROFSs:

26 $${Q}_{G}=\left\{\langle {\vartheta ,\mu }_{{Q}_{G}}(\vartheta ),{n}_{{Q}_{G}}(\vartheta )\rangle /\vartheta \epsilon U\right\}$$

27 $$0\le {\left({\mu }_{{Q}_{G}}(\vartheta )\right)}^{q}+{\left({n}_{{Q}_{G}}(\vartheta )\right)}^{q}\le 1, q\ge 1$$

SWARA weights different criteria based on their essence. A relation matrix is created in the first step in Eq. (28) (Ghenai et al. 2020):

28 $$Q_{k} = \left[ {\begin{array}{*{20}l} 0 \hfill & {Q_{12} } \hfill & \cdots \hfill & \cdots \hfill & {Q_{1n} } \hfill \\ {Q_{21} } \hfill & 0 \hfill & \cdots \hfill & \cdots \hfill & {Q_{2n} } \hfill \\ \vdots \hfill & \vdots \hfill & \ddots \hfill & \cdots \hfill & \cdots \hfill \\ \vdots \hfill & \vdots \hfill & \vdots \hfill & \ddots \hfill & \vdots \hfill \\ {Q_{n1} } \hfill & {Q_{n2} } \hfill & \cdots \hfill & \cdots \hfill & 0 \hfill \\ \end{array} } \right]$$

Next, the values of ${s}_{j}$, ${k}_{j}$, ${q}_{j}$, and ${w}_{j}$ are computed with Eqs. (29)–(31) (Rani et al. 2020):

29 $${k}_{j}=\left\{\begin{array}{c}1 j=1\\ {s}_{j}+1 j>1\end{array}\right.$$

30 $${q}_{j}=\left\{\begin{array}{c}1 j=1\\ \frac{{q}_{j-1}}{{k}_{j}} j>1\end{array}\right.$$

$$\mathrm{If }{s}_{j-1}={s}_{j}, {q}_{j-1}={q}_{j}$$; $$\mathrm{If }{s}_{j}=0, {k}_{j-1}={k}_{j}$$

31 $${w}_{j}=\frac{{q}_{j}}{\sum_{k=1}^{n}{q}_{k}}$$

Within this context, ${s}_{j}$ is the comparative importance rate, ${k}_{j}$ is the coefficient value, ${k}_{j}$ is the recalculated weight, and ${w}_{j}$ represents the weights. In addition, the values are limited and transposed to the power of 2t + 1.

## Results

### Illustrating innovative changes

The first step is modeling the total e-trade volume in the USA uploaded from FRED. The paper uses a long data period from January 1996 to February 2021 to detect the model with the best accuracy.

**Fig. 1:**
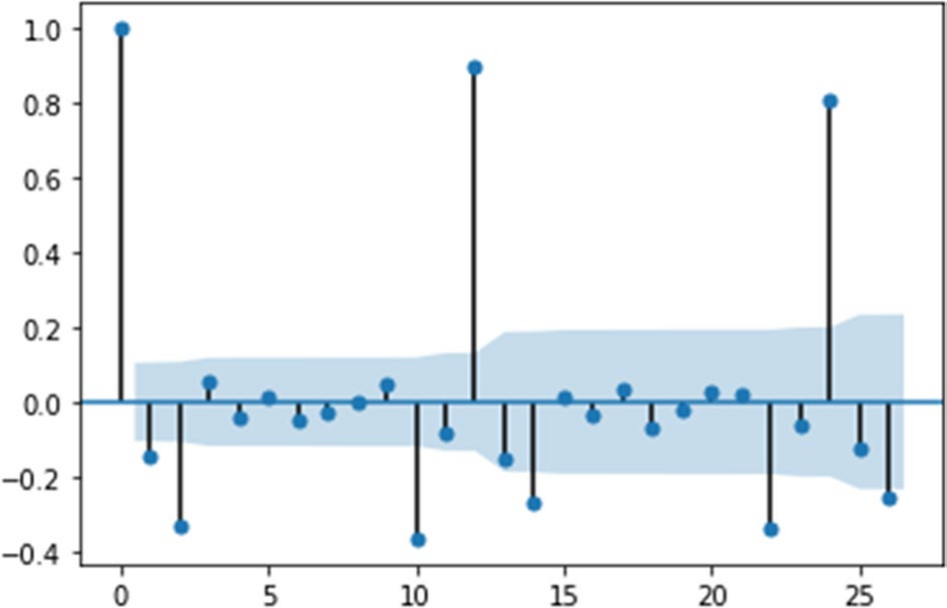
Autocorrelation function for US e-trade volume from 1 to 25 years

The analysis covers monthly data from January 1996 to February 2021. Figure 1 presents the autocorrelation function (ACF) for percentage changes in the original time series.

Table 1 shows that the initial choice of ACF specification is beneficial since all the coefficients show high significance. Thus, this specification can be a base for building a TVP model.

**Table 1:** Model summary for US e-trade volume

Parameter	Coef	Std err	z	*P* >|z|	[0.025	0.975]
Intercept	0.0024	0.001	1.831	0.067	− 0.000	0.005
ar.L1	− 0.5714	0.042	− 13.601	0.000	− 0.654	− 0.489
ar.L2	− 0.3695	0.042	− 8.773	0.000	− 0.452	− 0.287
ar.S.L12	0.9477	0.011	83.587	0.000	0.925	0.970
Sigma2	0.0020	0.000	17.472	0.000	0.002	0.002

The second column (Coefficient) of Table 1 shows the values of the model’s coefficients plus the magnitude of the model’s variance (sigma2), the third column (SE) corresponds to the parameters’ standard error, the fourth column (z) displays z-statistics, needed for determining the significance of coefficients, the fifth column (P) denotes the *p*-value for each coefficient that can be interpreted as its significance, the sixth ([0.225) and the seventh (0.975]) columns show the lower and upper bound for 95% confidence interval for computed coefficients.

TVP model for US e-trade volume will look as follows:

32 $${y}_{t}={\beta }_{0,t}+{\beta }_{1,t}{y}_{t-1}+{\beta }_{2,t}{y}_{t-2}+{\beta }_{3,t}{y}_{k,t-12}+{\nu }_{t}, {\nu }_{t}\sim N\left(0,{\sigma }_{\nu }^{2}\right)$$

where ${\beta }_{i,t}$ are modeled as shown in (2).

**Fig. 2:**
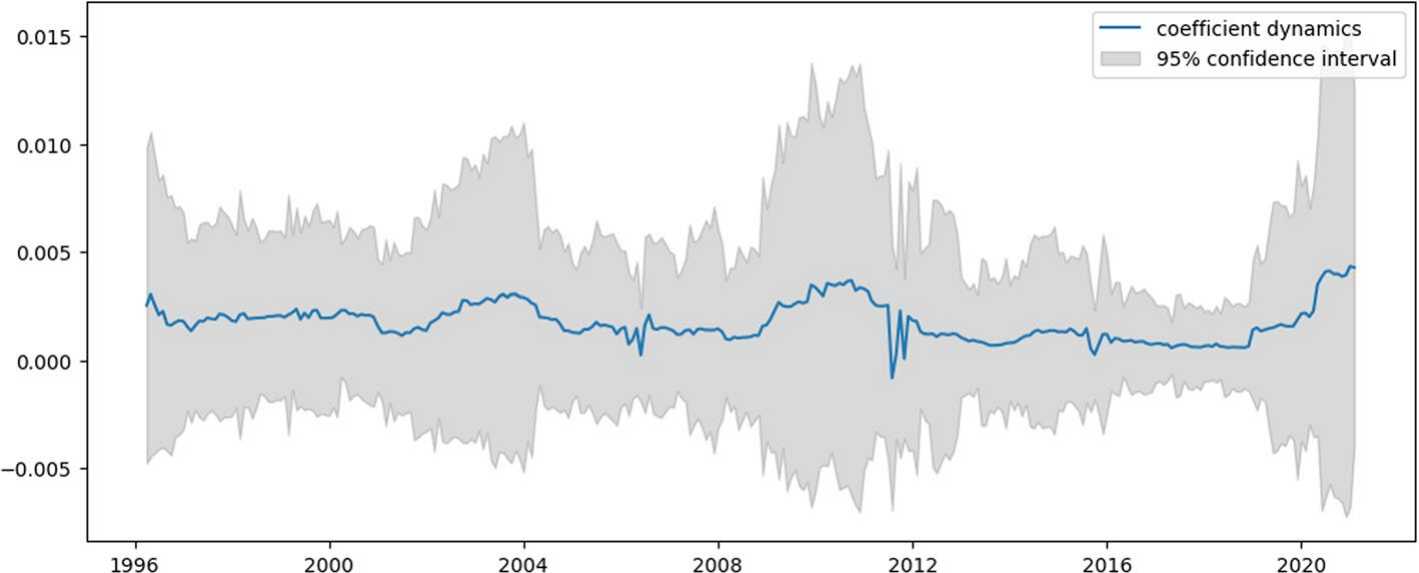
Intercept dynamics for US e-trade volume

**Fig. 3:**
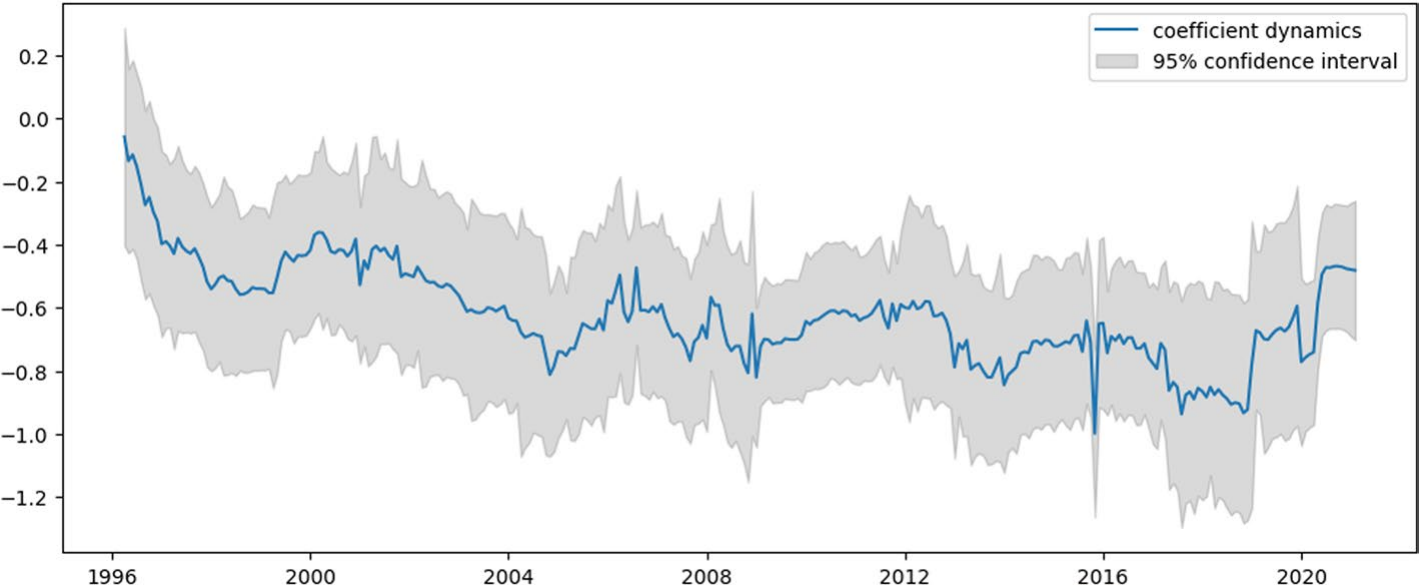
${{\varvec{\beta}}}_{1,{\varvec{t}}}$ dynamics for US e-trade volume

**Fig. 4:**
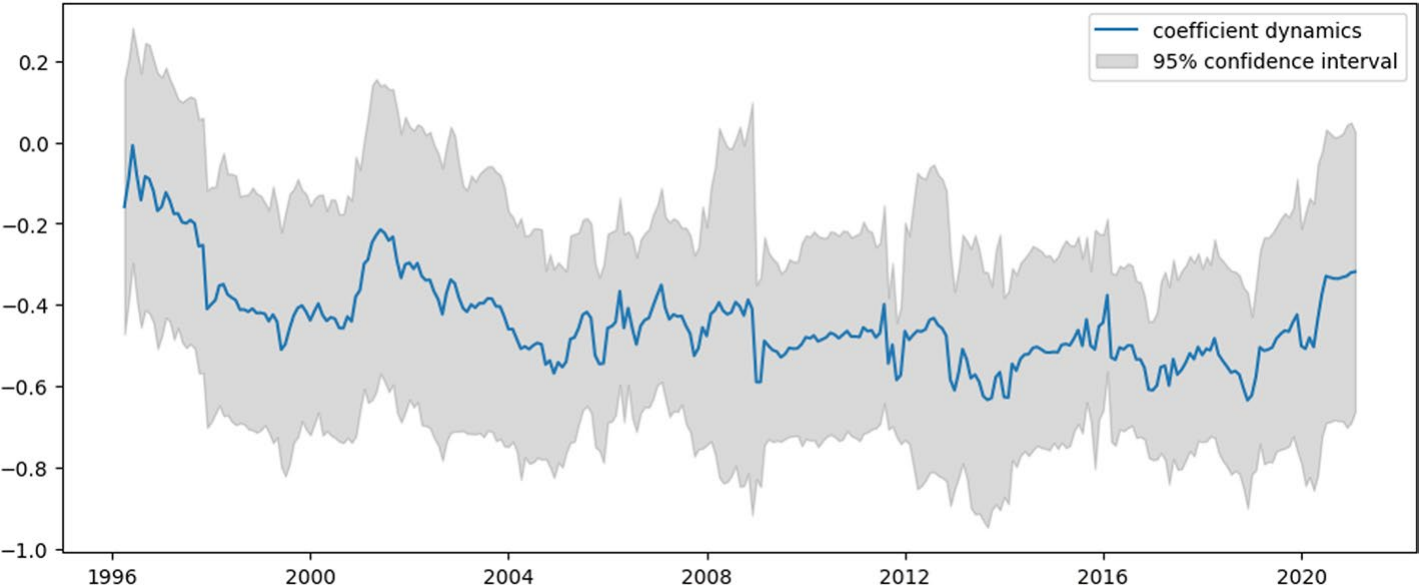
${{\varvec{\beta}}}_{2,{\varvec{t}}}$ dynamics for US e-trade volume

**Fig. 5:**
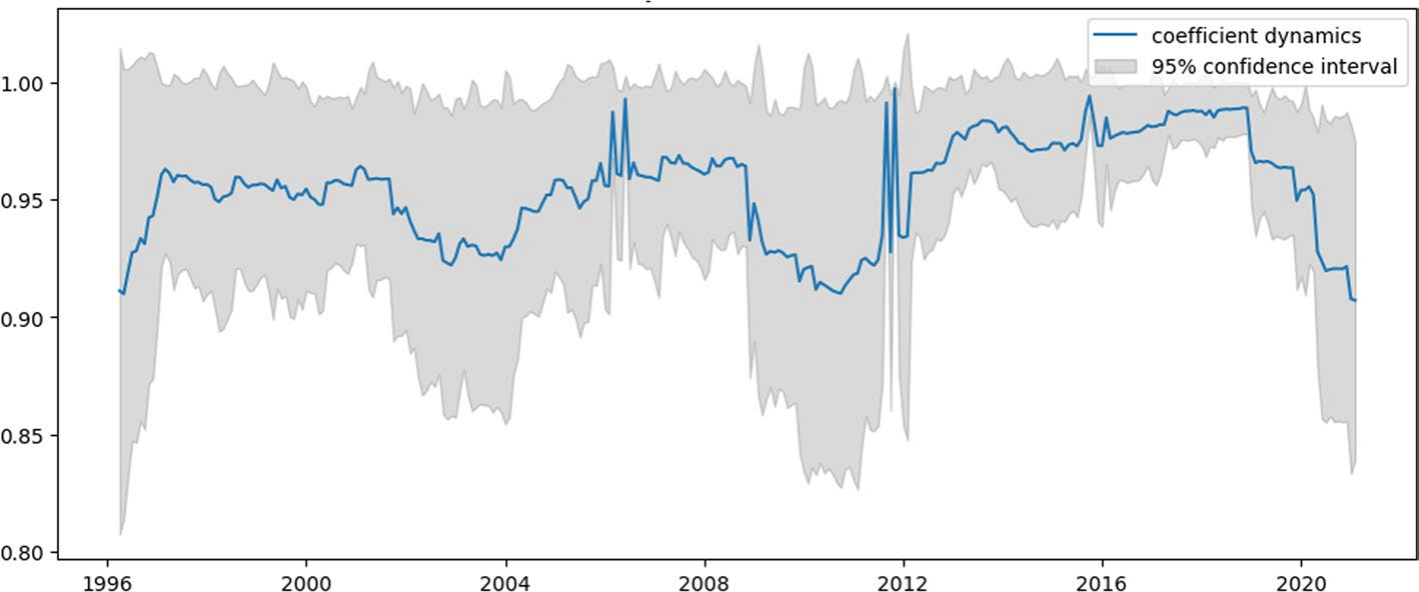
${{\varvec{\beta}}}_{3,{\varvec{t}}}$ dynamics for US e-trade volume

Figures 2, 3, 4 and 5 present the dynamics of all four coefficients of Eq. (32) computed by the TVP regression methodology described in the previous section. The dynamics of the intercept are analyzed first (Fig. 2).

This figure tracks three periods of intercept growth, which can be interpreted as an acceleration phase in e-trade expansion. The first period begins in the early 2000s and coincides with the dot-com crisis. The second period begins in 2008 and coincides with the substandard mortgage financial crisis. The third period begins in 2019, corresponding to the decline in major US stock indices, which is then accelerated by 2020. The revealed relation of humps in Fig. 2 to stock market behavior is not a coincidence. Indeed, people tend to switch to online shopping when disposable income falls since it allows for the most lucrative offers to be found. Naturally, uncertainty about the true value of the intercept lengthens the time of the mentioned crises. However, based on its expected value, conclusions may still be drawn. It is worth noting that the constructed model indicates when the unprecedented acceleration of e-trade growth in the USA begins.

Examining Figs. 3 and 4, which represent the dynamics of AR1 and AR2 in Eq. (32), these coefficients show similar dynamics, mostly for the negative values. Apart from intercept dynamics, these two autoregressive coefficients do not indicate the abovementioned economic crises. Recently, they both experienced quite significant growth, which can be interpreted as this process becoming less inertial.

Analyzing Fig. 5, which shows the dynamics of the AR12 coefficient, it can be concluded that it is very closely negatively connected to the dynamics of the intercept. The periods of troughs in this graph correspond to a reduction in the seasonality component in the series. Again, these periods are associated with higher volatility. Table 2 compares constructed TVP and trivial model (mean of percentage changes) efficiencies.

**Table 2:** Model efficiency summary for US e-trade volume

MSFE TVP	MSFE trivial	MAE TVP	MAE trivial	RMSE TVP	RMSE trivial
0.0016	0.02	0.0412	0.1423	0.0394	0.1419

**Fig. 6:**
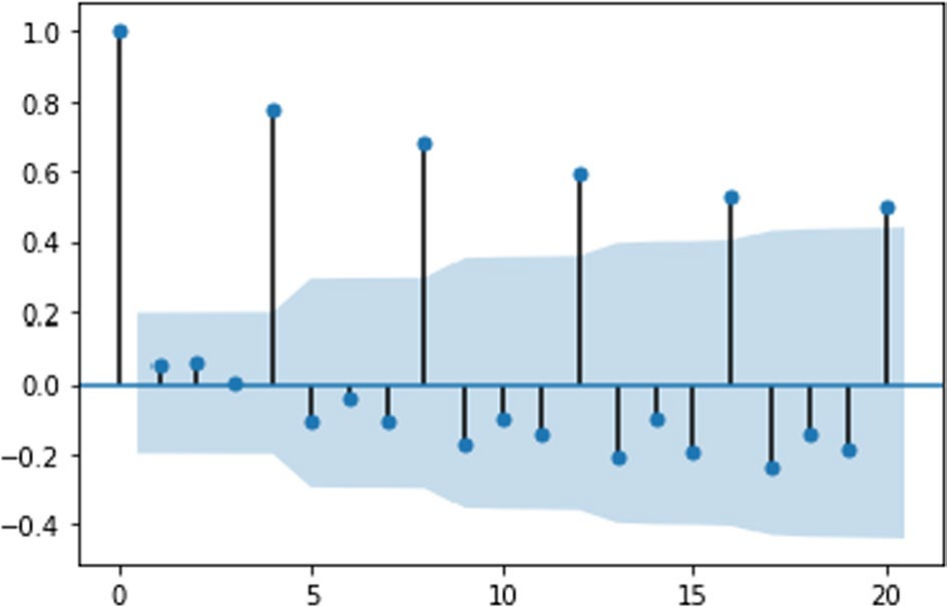
Autocorrelation function for Amazon revenue

The model significantly outperforms the trivial model, judging by mean squared forecast error (MSFE), root-mean-squared error (RMSE), and mean absolute error (MAE). Thus, it is considered quite reliable (Table 2). As a result, having analyzed the USA e-trade volume dynamics based on TVP regression, it can be confirmed that shock effects contributed to US e-commerce development while decreasing its seasonality and inertia components. The two largest e-trade platforms in the world are Amazon.com, Inc. and Alibaba Group. The paper will analyze their revenues by the same methodology as above. The analysis aims to evaluate whether shock effects also affect performance similar to effects on the observed total US e-trade volume. The analysis starts with Amazon, with data uploaded from YCharts.com. The analysis covers quarterly data from 1Q 2004 to 2Q 2021. Figure 6 presents the ACF for percentage changes in the original time series.

Table 3 shows that the initial choice of the model specification is favorable since the AR4 term is highly significant. Concerning the intercept—it will be kept despite insignificance to analyze the change in the speed of Amazon’s revenue growth. Thus, the study takes this specification as the base for building the TVP model.

**Table 3:** Model summary for Amazon revenue

Parameter	Coef	St. error	z	*P* >|z|	[0.025	0.975]
Intercept	0.0208	0.023	0.917	0.359	− 0.024	0.065
ar.S.L4	0.9514	0.033	29.214	0.000	0.888	1.015
Sigma2	0.0223	0.002	11.522	0.000	0.019	0.026

TVP model for Amazon revenue will look as follows:

33 $${y}_{t}={\beta }_{0,t}+{\beta }_{1,t}{y}_{t-4}+{\nu }_{t}, {\nu }_{t}\sim N\left(0,{\sigma }_{\nu }^{2}\right)$$

where ${\beta }_{i,t}$ are modeled as in (2).

**Fig. 7:**
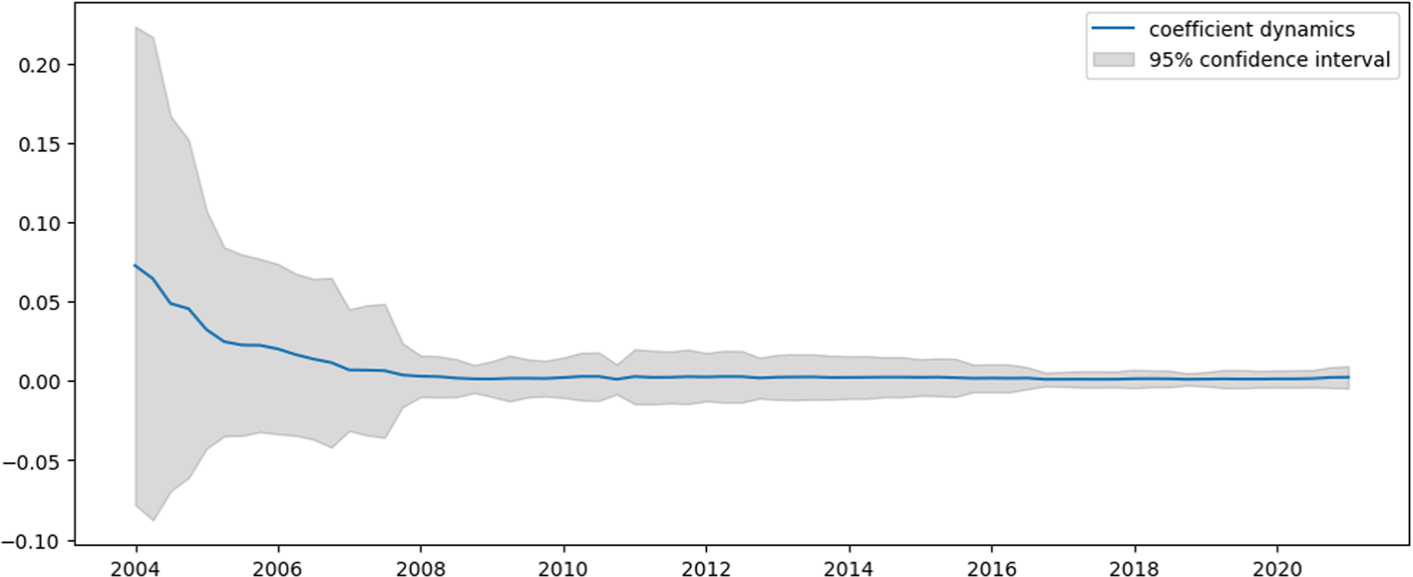
Intercept dynamics for Amazon revenue

**Fig. 8:**
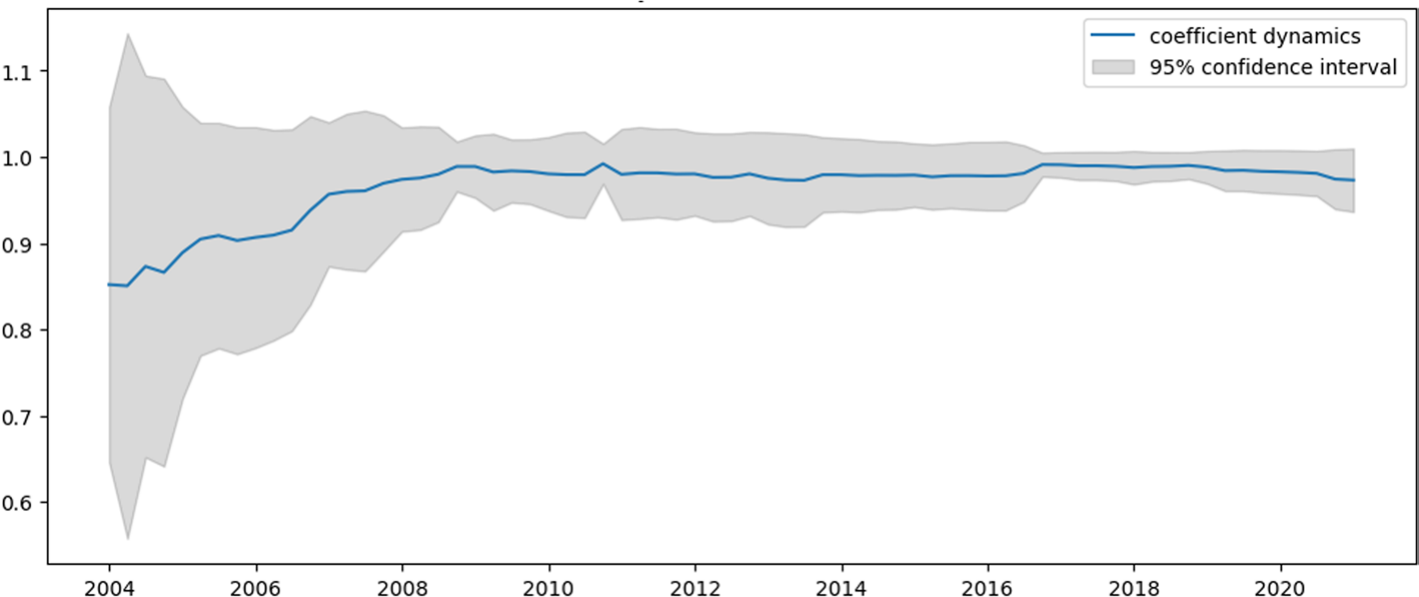
${{\varvec{\beta}}}_{1,{\varvec{t}}}$ dynamics for Amazon revenue

Figures 7 and 8 present the dynamics of coefficients for Eq. (33), computed by the TVP regression methodology described in the previous section. These graphs indicate that the study does not track any correspondence to US recession periods. It can detect any significant influence of shock effects. Both intercept and AR4 coefficients are stable in time except for the period from 2004 to 2007, where revenue dynamics experienced an increase in the seasonality component and a decrease in the average growth rate. This phenomenon may be connected to the dot-com crisis in the early 2000s. However, since that period, Amazon has developed quite stably, and thus far, significant shock effects have not been revealed.

Table 4 compares constructed TVP and trivial model (mean of percentage changes) efficiencies.

**Table 4:** Model efficiency summary for Amazon revenue

MSFE TVP	MSFE trivial	MAE TVP	MAE trivial	RMSE TVP	RMSE trivial
0.0086	0.13	0.0799	0.36	0.0825	0.382

**Fig. 9:**
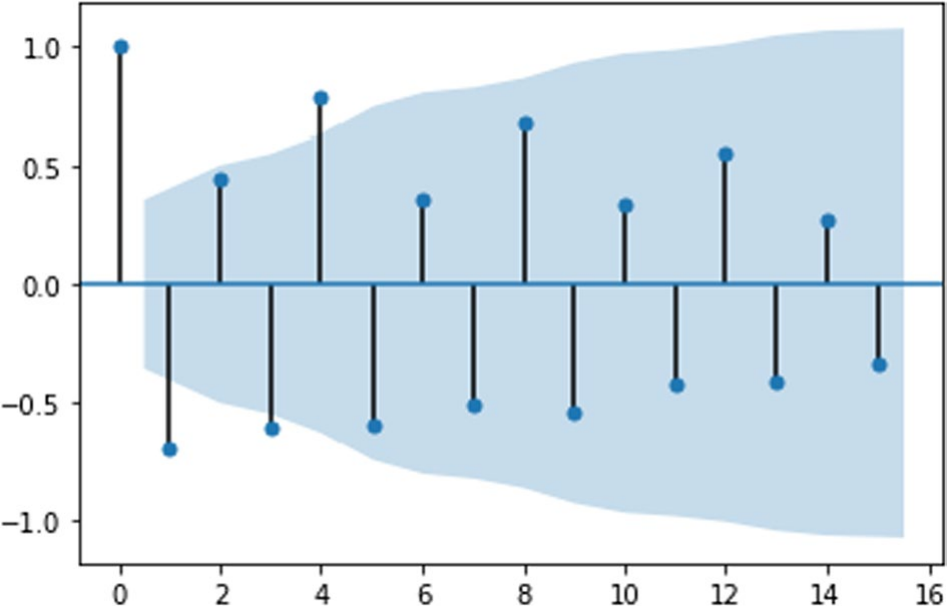
Autocorrelation function for Alibaba revenue

The TVP model is significant, as in Table 2, and outperforms the trivial model, as judged by MSFE, RMSE, and MAE. Thus, it can be considered sufficiently reliable. Now the study moves on to the data of Alibaba, also uploaded from YCharts.com. The analyzed data set covers monthly data from 1Q 2016 to 2Q 2021. Figure 9 presents the ACF for percentage changes in the original time series.

Table 5 shows that the initial choice of the model specification is also favorable since the AR4 term is highly significant. Concerning the intercept—it will also be kept despite insignificance to analyze the change in the speed of Alibaba’s revenue growth. Thus, it will take this specification as a base for building a TVP model.

**Table 5:** Model summary for Alibaba revenue

Parameter	Coef	Std err	z	*P* >|z|	[0.025	0.975]
intercept	0.0047	0.006	0.735	0.462	− 0.008	0.017
ar.S.L4	0.9690	0.025	39.381	0.000	0.921	1.017
sigma2	0.0070	0.002	3.271	0.001	0.003	0.011

TVP model for Alibaba revenue will have the same specification as in (13).

**Fig. 10:**
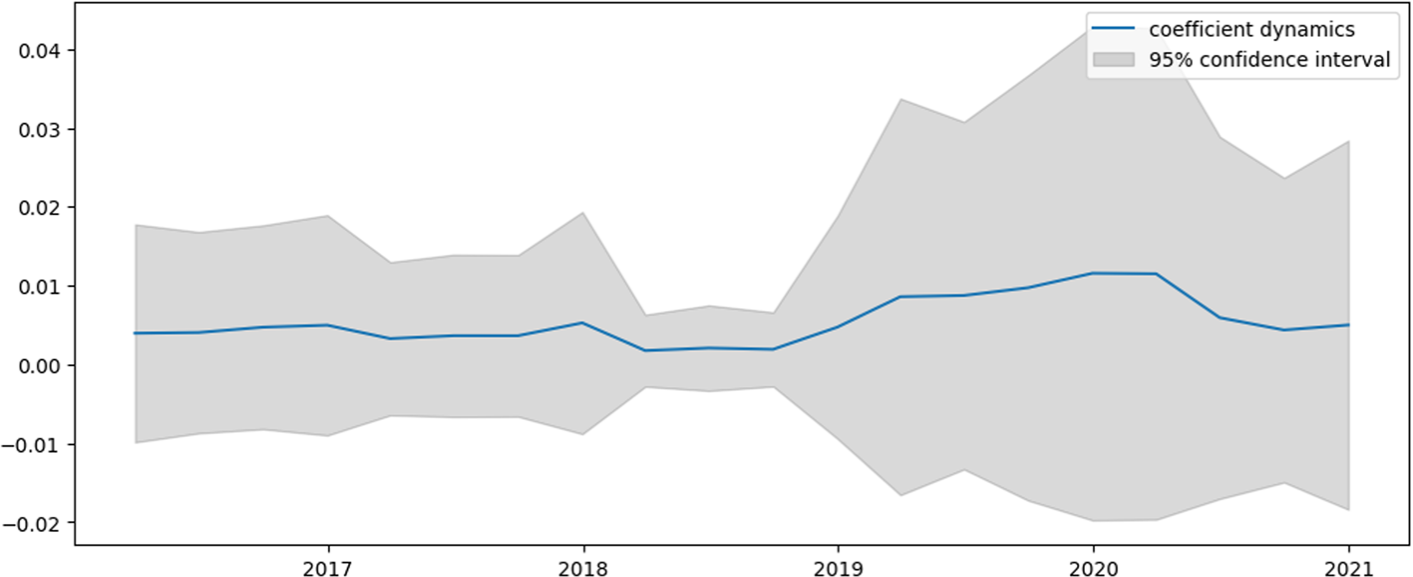
Intercept dynamics for Alibaba revenue

**Fig. 11:**
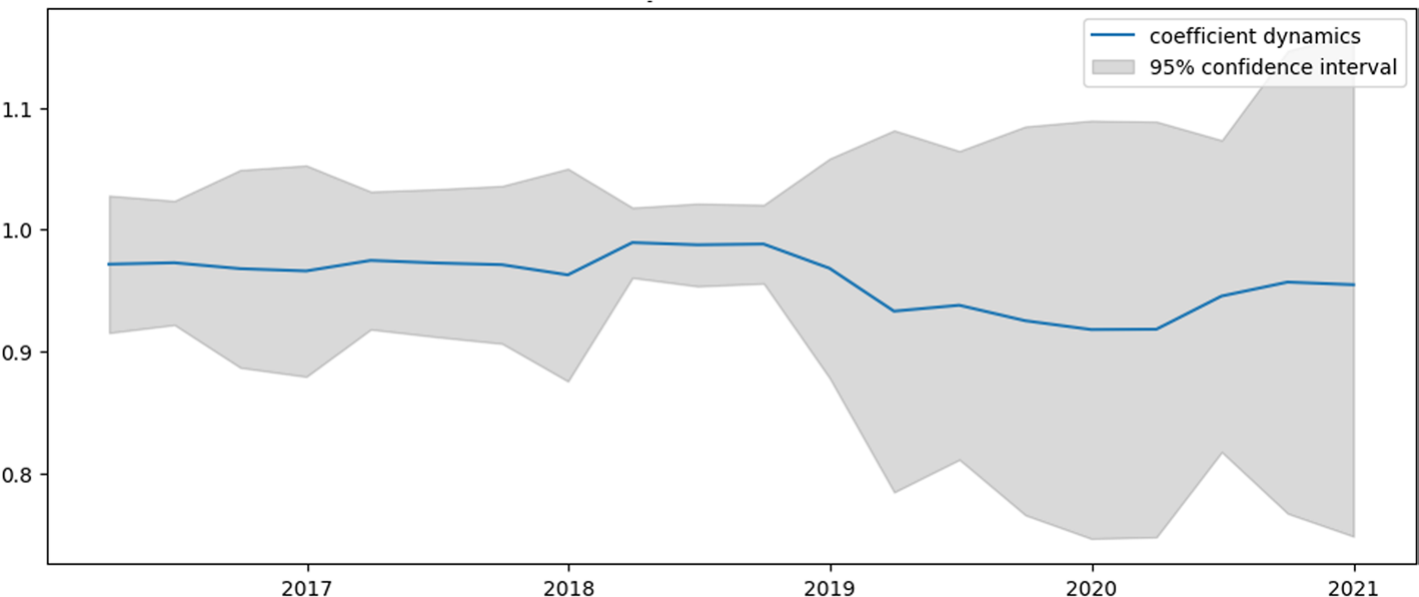
${{\varvec{\beta}}}_{1,{\varvec{t}}}$ dynamics for Alibaba revenue

Figures 10 and 11 present the dynamics of coefficients computed by the TVP regression methodology described in the previous section.

As can be seen from these graphs, 2019 and 2020 are characterized by increased uncertainty about the true parameter values. The intercept is the highest in the second quarter of 2020 but returns to the average level after that. As for the AR4 term, since the pandemic’s outbreak, Alibaba's revenue dynamics have become a slightly stronger seasonality component (Fig. 11). In any case, the paper cannot state that the shocks significantly affected Amazon’s revenue. Table 6 compares the constructed TVP and trivial (mean of percentage changes) model efficiencies.

**Table 6:** Model efficiency summary for Alibaba revenue

MSFE TVP	MSFE trivial	MAE TVP	MAE trivial	RMSE TVP	RMSE trivial
0.0062	0.0922	0.0772	0.3037	0.0854	0.3211

**Fig. 12:**
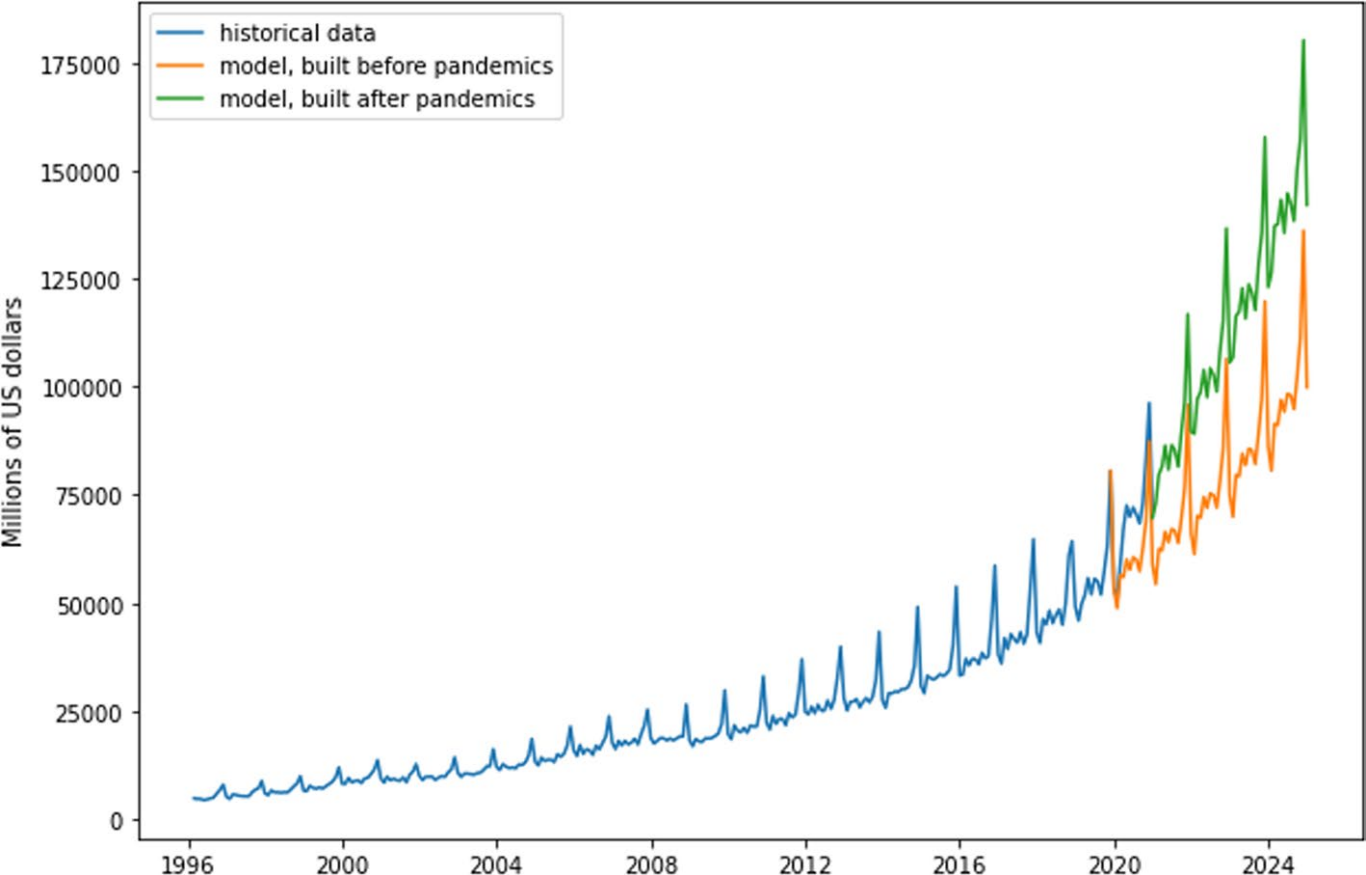
Effect forecast and US e-trade sector revenue (USD, millions)

TVP model efficiency is significantly similar to that shown in Tables 2 and 4 and outperforms the trivial model, as judged by MSFE, RMSE, and MAE. Thus, it can be considered sufficiently reliable. The situation reveals significant shock effects on total US e-trade volume but no statistically significant effects on Amazon and Alibaba revenue. We thus conclude that this additional acceleration in e-trade happened because other companies began to offer and promote online services and platforms. Therefore, growing competition can be expected in this market, which will probably end the hegemony of current e-trade giants. To quantify shock effects on US e-trade, the paper compares forecasts obtained by applying the predicted parameter states of two models: one built before the coronavirus outbreak and the other after the outbreak. Thus, the first model is based on statistical information from January 1996 to December 2019, whereas the second uses data from January 1996 to February 2021. The paper uses predicted parameter states with the intent of obtaining forecasts for January 2025. Figure 12 presents the results of the modeling.

The difference between the green and the orange lines shows shock effects over time. E-trade volume in the USA is projected to be approximately 35% higher by 2025 than it would have been without the pandemic effect.

### Analyzing balanced scorecard-based open financial innovation models for the e-commerce industry

Open financial innovation models are selected for the e-commerce industry based on balanced scorecard perspectives. Information regarding the operationalization of the variables is detailed in Table 7.

**Table 7:** Balanced scorecard-based open financial innovation models

Models	References
Financial institution-oriented financial facilities (Model 1)	Alam et al. (2019); Dabrowski and Lottermoser (2019)
Customer interaction in financial issues (Model 2)	Poon et al. (2020); Al-Dmour et al. (2020)
Learning of new financial ideas with competition and benchmarking (Model 3)	Chen (2018); Xiao and Ke (2021)
Organizational excellence in collaborative financial ideas (Model 4)	Liu et al. (2021); Meng et al. (2021)

Financial innovation models can be based on financial institution-oriented financial facilities. Additionally, customer interactions about financial issues can be considered. Moreover, the financial innovation model can include learning new financial ideas with competition and benchmarking. Finally, organizational excellence in collaborative financial ideas can play a critical role in this issue. Table 8 values are used in the calculation process.

**Table 8:** Scales and degrees

Linguistic Scales for Criteria	Membership degrees	Non-membership degrees
No influence (n)	0.40	0.25
somewhat influence (s)	0.45	0.28
medium influence (m)	0.50	0.31
high influence (h)	0.55	0.34
very high influence (vh)	0.60	0.37

In this scope, three decision makers make evaluations. These experts have significant experience regarding the open financial innovation models for e-commerce industry. Table 9 includes the evaluations.

**Table 9:** Linguistic evaluations

	M1	M2	M3	M4
*Decision maker 1*				
M1		S	VH	M
M2	M		S	VH
M3	H	M		M
M4	M	VH	VH	
*Decision maker 2*				
M1		H	VH	H
M2	M		S	VH
M3	H	M		M
M4	H	VH	H	
*Decision maker 3*				
M1		M	VH	H
M2	S		VH	VH
M3	H	M		M
M4	N	VH	H	

Table 10 includes average values.

**Table 10:** Average values

	M1		M2		M3		M4	
	μ	v	μ	v	μ	v	Μ	v
M1			0.50	0.31	0.60	0.37	0.53	0.33
M2	0.48	0.30			0.50	0.31	0.60	0.37
M3	0.55	0.34	0.50	0.31			0.50	0.31
M4	0.48	0.30	0.60	0.37	0.57	0.35		

Table 11 demonstrates the score function values.

**Table 11:** Score function values

	M1	M2	M3	M4
M1	0.000	0.095	0.165	0.116
M2	0.086	0.000	0.095	0.165
M3	0.127	0.095	0.000	0.095
M4	0.086	0.165	0.139	0.000

Table 12 includes the significant values used in the analysis process.

**Table 12:** Sj, kj, qj, and wj values

M1	Sj	kj	qj	wj	M2	Sj	kj	qj	wj
M3	0.165	1.000	1.000	0.368	M4	0.165	1.000	1.000	0.363
M4	0.116	1.116	0.896	0.330	M3	0.095	1.095	0.913	0.332
M2	0.095	1.095	0.818	0.301	M1	0.086	1.086	0.840	0.305
M3	Sj	kj	qj	wj	M4	Sj	kj	qj	wj
M1	0.127	1.000	1.000	0.354	M2	0.165	1.000	1.000	0.372
M2	0.095	1.095	0.913	0.323	M3	0.139	1.139	0.878	0.327
M4	0.095	1.095	0.913	0.323	M1	0.086	1.086	0.808	0.301

Relation matrix is constructed in Table 13.

**Table 13:** Relation matrix

	M1	M2	M3	M4
M1		0.301	0.368	0.330
M2	0.305		0.332	0.363
M3	0.354	0.323		0.323
M4	0.301	0.372	0.327	

Table 14 explains the stable matrix.

**Table 14:** Stable matrix

	M1	M2	M3	M4
M1	0.243	0.243	0.243	0.243
M2	0.250	0.250	0.250	0.250
M3	0.255	0.255	0.255	0.255
M4	0.253	0.253	0.253	0.253

**Fig. 13:**
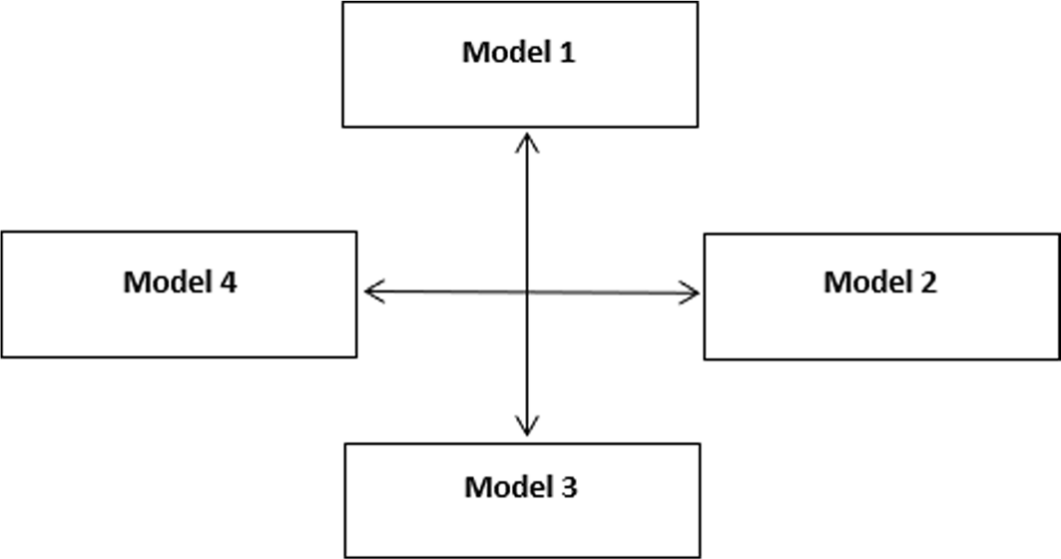
Impact-relation map for the models

Causal relationship results are illustrated in Fig. 13.

Figure 13 demonstrates a mutual relationship between financial institution-oriented financial facilities (Model 1) and learning new financial ideas with competition and benchmarking (Model 3). This situation is also appropriate for customer interactions in financial issues (Model 2) and organizational excellence in collaborative financial ideas (Model 4). In addition to q-ROFSs, weights are computed with IFSs and PFSs. Comparative results are provided in Table 15.

**Table 15:** Comparative model weighting priorities

	IFSs	PFSs	q-ROFSs
M1	4	4	4
M2	3	3	3
M3	2	1	1
M4	1	2	2

The results prove the ideas from the literature discussed in the literature review for e-commerce, open innovation, market capitalization shocks, and methods. PFS and q-ROFS results identify learning new financial ideas with competition and benchmarking (Model 3) as the most crucial mode for open financial innovation models in the e-commerce industry. On the other hand, organizational excellence in collaborative financial ideas (Model 4) is the most significant model according to the calculations with IFSs. The rankings of the third and fourth models are the same in all fuzzy sets. The conclusions differ from those of existing studies: the quality, momentum, cost, and size of companies in the US e-commerce sector can lead to varying intensities for the role of market capitalization in open innovation models. It has historically performed via shock effects during the various stages of the economic cycle and underlying factors. For investors wishing to capitalize on the potential benefits of the role of market capitalization in open innovation models, many studies have confirmed the effect (Samaha et al. 2014; Lee et al. 2018; Yigitcanlar et al. 2020; Tayal et al. 2021; Hermawati et al. 2020). Comparative model weighting priorities recommend learning new financial ideas with competition and benchmarking (M3) and explaining the phenomenon of the study.

## Discussion

Conceptually, this paper depends on business models based on open innovation to solve the problem of increased innovation-creation costs using external R&D resources and stimulate economic growth as expansion occurs in the markets using these models. In open innovation models, intellectual property is not protected from competitors but rather treated as a commodity to be actively exchanged with other subjects of the innovation, as well as other counteragents (Yigitcanlar et al. 2020). This perspective generates many advantages for various enterprises (Park and Choi 2019; Povolna 2019; Poon et al. 2020). Importantly, the role of market capitalization in the open innovation effect on big-cap companies is weaker than for small-capitalization companies in the US e-commerce sector. This arises from the positive impact of spread production networks within shock conditions. The multifactor ecosystem effects of the shocks are based on a long-standing concept of diversification. The combination of the ecosystem effects of several factors of big-cap companies is not the same as for small-cap companies (Dvoulety 2019; Cooke 2020).

This paper supports the advent of advanced TVP that allows factor risks to be scientifically integrated into the multifactor ecosystem (Zhang et al. 2019; Chao et al. 2020; Li et al. 2021; Kou et al. 2022; Chao et al. 2022; Li et al. 2022). This model assumes that each share has a certain level of sensitivity to the movement of the role of market capitalization for open innovation models on company revenue. This specifically features a basic factor model that assumes one factor—other countries’ capitalizations—drive the revenue of companies in the US e-commerce sector. This research contributes to studies about the organization of e-commerce open innovation in the USA for other excellence (Germann et al. 2015; Moorman and Day 2016; Wedel and Kannan 2016). This paper also develops ideas of sustainability in marketing research and technological open innovation process in the USA based on value cocreation and can be divided into several types (Iansiti and Levien 2004; Gawer 2014; Chang et al. 2015; Buhalis and Foerste 2015). This situation is also appropriate for customer interactions in financial issues and organizational excellence in collaborative financial ideas. The findings also demonstrate that learning new financial ideas with competition and benchmarking is the most significant mode for open financial innovation models in the e-commerce industry based on the results with PFSs and q-ROFSs. Furthermore, organizational excellence in collaborative financial ideas is the most significant model regarding the calculations with IFSs. The rankings of the third and fourth models are the same in all fuzzy sets (Alagidede and Ibrahim 2017; Khushboo and Syeedun 2019).

The novelty of this research is using new data to make a detailed analysis and forecast of e-commerce companies in the USA and the associated macroeconomic ecosystem effects. Another novelty of this study is making evaluations using an econometric model with a fuzzy decision-making methodology. This situation provides the opportunity for more precise results. Furthermore, appropriate strategies can be created to improve open financial innovation models for the e-commerce industry. These findings add to the growing literature on the role of market capitalization in open innovation models of the e-commerce sector (Hansen and Seo, 2002; Agarwal 2021). Future research can explain how production network spread impacts e-commerce networks exactly.

## Conclusions

The article features existing dependencies between the future development of e-trade in the USA with shock effects and the future development of e-trade without shock effects. These factors have been studied using tools of state of space analysis, as well as graphical analysis and model criteria analysis. Hence, the majority of e-trade considered open innovation development a way to both minimize environmental impacts and ensure economic stability and sustainability during the COVID-19 pandemic. Thus, this paper determined the financial parameters of the US e-commerce sector. The relationship between company revenue forecasts based on prepandemic and postpandemic indicators is proposed as the indicator of the role of market capitalization in open innovation models. On the other hand, an evaluation is performed that considers a fuzzy decision-making methodology. For this purpose, balanced scorecard-based open financial innovation models for the e-commerce industry are weighted with an M-SWARA method based on q-ROFSs and the golden cut. The conclusion is that a mutual relationship exists between financial institution-oriented financial facilities and learning new financial ideas with competition and benchmarking.

## Strengths and limitations

A strength of this study is the use of new data to conduct a detailed analysis and forecast for e-commerce companies in the USA and the associated macroeconomic ecosystem effects on Amazon and Alibaba. This study has potential limitations: (1) the choice of only two e-commerce companies as benchmarks for all markets; (2) the use of a long data period ranging from January 1996 to February 2021. A shorter data period could provide different results.

Future research could ensure the credibility and reliability of the developed model by implementing data decomposition approaches. The comments presented above about novelty are valid for analyzing the current state. Upon a deeper analysis of the current state, these comments should be eliminated.

## Practical and theoretical implications

The contribution of the research is that the structure of the e-commerce sector in China and the USA is complex, with weaker shock effects for small-market-capitalization companies (market capitalizations from $300 million to $2 billion). The contribution to the growing literature on the effects of market capitalization on open innovation models in the e-commerce sector is based on new data for a detailed analysis and forecast of e-commerce companies in the USA and the associated macroeconomic ecosystem effects.

## Data Availability

In this study, the data available at Mendeley Data: Mikhaylov, A. 2021. US e-commerce: COVID-19 Effect on Alibaba and Amazon, Mendeley Data, V1, https://doi.org/10.17632/bcjwyrdr57.1
